# Nanocatalysis for Environmental Protection, Energy, and Green Chemistry

**DOI:** 10.3390/nano13222958

**Published:** 2023-11-16

**Authors:** Paraskevi Panagiotopoulou, Ioannis V. Yentekakis

**Affiliations:** 1 Laboratory of Environmental Catalysis, School of Chemical and Environmental Engineering, Technical University of Crete (TUC), GR-73100 Chania, Greece; ppanagiotopoulou@tuc.gr; 2 Laboratory of Physical Chemistry and Chemical Processes, School of Chemical and Environmental Engineering, Technical University of Crete (TUC), GR-73100 Chania, Greece; 3 Foundation for Research and Technology-Hellas/Institute of GeoEnergy (FORTH/IG), GR-73100 Chania, Greece

## 1. General Remarks

Nowadays, nanoscience and nanotechnology depict cutting-edge areas of modern science and technology across an array of applications, including heterogeneous catalysis. The latter is often called “nanocatalysis”, describing the consensus that the design of nano-structured catalysts prevail in modern fundamental and applied catalysis [1,2,3,4,5]. The rational design of nanostructured catalysts, involving the catalyst’s active phases, possible promoters, and support materials, provides composite materials characterized by well-tuned activity/selectivity/stability in applications related to environmental protection and remediation, circular economy, sustainability, and green energy technologies, as well as green chemicals production.

MDPI’s journal “Nanomaterials” has become among the most highly regarded journals in the field to serve as a platform for innovative results and ideas to stimulate and discuss scientific research in the aforementioned topics and related fields, providing high readership and reliability.

This Special Issue, entitled “Nanocatalysis for Environmental Protection, Energy, and Green Chemistry”, was aimed to host significant advances in these areas mainly collected from, but not limited to, works presented in the “16th Panhellenic Symposium of Catalysis” (https://www.16psc.tuc.gr (accessed on 9 November 2023)), which were chaired by us (I.V.Y. and P.P.). Since its first organization in 1987, the Panhellenic Symposium of Catalysis (PSC) has been established as among the highest quality scientific meetings in Greece and Cyprus, being held every two years and organized by the Greek Catalysis Community. The latter lists approximately 400 highly active members who strongly and distinctly contribute to the development of catalysis science in all its directions and cutting-edge topics.

In this context, this Special Issue compiled 11 high-quality contributions [6,7,8,9,10,11,12,13,14,15,16] (10 collected from PSC participants and 1 from others), covering recent research progress in the titled theme. These articles are briefly discussed below.

## 2. Contributions and Highlights of this Special Issue

Zaspalis and co-workers [6] synthesized La_1−x_Sr_x_MnO_3±δ_ and La_1−x_Ca_x_MnO_3±δ_ (x = 0–0.5) perovskites and studied their performance in a chemical looping process using CH_4_ as fuel. La_1−x_Sr_x_MnO_3±δ_ and La_1−x_Ca_x_MnO_3±δ_ perovskites exhibited identical defect chemistry obtaining both an excess and a deficiency of oxygen; the oxygen excess increased as x and temperature decreased, while the oxygen deficiency increased as x and temperature increased. The relation between the defect chemistry of the materials and their behavior in chemical looping processes was determined. Their oxygen transfer capacity under reductive conditions is composed by two distinct reduction reactions that produce oxygen vacancies: the Mn^4+^ → Mn^3+^ reduction drives the CH_4_ oxidation to CO_2_, while the Mn^3+^ → Mn^2+^ reduction drives it to CO. That is, the fuel may react with various types of oxygen molecules available within the materials, which are generated by different mechanisms. The relative amounts of each oxygen type determine the CO_2_/CO selectivity and depend on the composition of materials as well as on the partial pressure of oxygen used for regenerating them.

Photocatalytic CO_2_ reduction was the topic of the work of Zindrou and Deligiannakis [7]. The authors elucidated that electron paramagnetic resonance (EPR) spectroscopy in combination with analytical anodic stripping voltammetry (ASV) can be a useful tool in aiding to quantitatively understand the solid–solution interface photocorrosion phenomena for the Cu_2_O photocatalyst. Cu_2_O is among the most promising photocatalysts for CO_2_ reduction; however, its photocorrosion remains a challenge.

Lymperi et al. [8] successfully used the electrochemical promotion of catalysis (EPOC) concept to improve the catalytic activity and selectivity of the CO_2_ hydrogenation reaction over a Pt catalyst film supported on an Yttria Stabilized Zirconia (YSZ) solid electrolyte. The as-constructed *CO_2_*, *H_2_*, *Pt/YSZ/Pt*, *air* electrochemical cell system was studied in two distinct operation modes: (i) when the necessary energy for the electrochemical promotion was produced through the parallel reaction of H_2_ oxidation (galvanic operation) and (ii) when a galvanostat/potentiostat was used to impose the necessary potential (electrolytic operation). The performance of the fuel cell (i) declined less than 15% in the presence of the reactant mixture (CO_2_ and H_2_) while producing enough current to conduct EPOC experiments. During the electrolytic operation of the electrochemical cell (ii), the CO production rate was increased by up to 50% due to the promotion of the reverse water–gas shift reaction.

The catalytic production of H_2_ from formic acid (FA) was reported by Loudoudi and co-workers [9]. The authors prepared three imidazole-based hybrid materials, coded as IGOPS, IPS, and impyridine@SiO_2_ nanohybrids via the covalent immobilization of N-ligands onto a mesoporous nano-SiO_2_ matrix. Their catalytic activity (TONs, TOFs), stability, and reusability were assessed for FA dehydrogenation. It was concluded that the low-cost imidazole-based nanohybrids IGOPS and IPS are capable of forming [Fe^2+^/IGOPS/PP_3_] and [Fe^2+^/IPS/PP_3_] heterogeneous catalytic systems with high stability and performance for FA dehydrogenation.

The production of synthesis gas (H_2_ + CO; a Fischer–Tropsch industry feedstock to produce value-added chemicals) through H_2_O and CO_2_ co-electrolysis was reported by Bimpiri et al. [10]. The effects of the H_2_O/CO_2_ ratio (= 0.5–2) in the feed on the electrochemical performance and the quality of the produced syngas (H_2_/CO ratio) under co-electrolysis conditions at 900 °C were investigated in an YSZ electrolyte-supported solid oxide cell with a La_0_._75_Sr_0_._25_Cr_0_._9_Fe_0_._1_O_3-δ_ (LSCF) as the fuel electrode and lanthanum strontium manganate (LSM) perovskite as the oxygen electrode. The mixed ionic-electronic conductivity of the LSCF perovskite allows the cell to operate well under both reducing and oxidizing environments. The presence and absence of H_2_ in the H_2_O/CO_2_ feed was also tested. H_2_ in the feed resulted in a higher open circuit voltage (OCV), a smaller iV slope and R_p_ values, and a significant effect on the H_2_/CO ratio of the produced syngas; however, the maximum current density remained unaffected. Remarkably, the performance of the LSCF perovskite fuel electrode is not compromised by the exposure to oxidizing conditions, showcasing that this class of electrocatalysts retains their reactivity in oxidizing, reducing, and humid environments.

The production of green diesel was the topic of the work of Nikolopoulos et al. [11]. They synthesized 60 wt.% Ni/alumina catalysts using two preparation methods (wet impregnation and co-precipitation) in order to study the effects of preparation methods on the catalytic efficiency concerning the transformation of sunflower oil into green diesel in a semi-batch reactor. The catalyst prepared via co-precipitation exhibited a higher specific surface area and a smaller mean crystal size of the nickel nanoparticle—factors justifying its better efficiency compared to that synthesized via wet impregnation. Optimization of the reaction conditions over the most active catalyst, prepared via co-precipitation, led to the complete transformation not only of the sunflower oil (edible oil) but also of the waste cooking oil (non-edible oil) into green diesel.

The upgrading process of biodiesel to green (renewable) diesel was reported by Fani et al. [12]. Natural mordenite originated from volcanic soils in Greek islands, was activated using a HCl solution followed by a NaOH solution, and was used as a support for preparing two metallic nickel catalysts (30 wt.% Ni), which were evaluated based on the aforementioned process. Double activation of natural mordenite optimized its supporting characteristics, finally resulting in a supported nickel catalyst with (i) an enhanced specific surface area and pore diameter facilitating mass transfer; (ii) an easier reducibility of nickel particles; (iii) an enhanced Ni^0^ dispersion and thus high active surface; (iv) a balanced population of moderate and strong acid sites; (v) a resistance to sintering; and (vi) low coking. Over this catalyst, the production of a liquid consisting of 94 wt.% renewable diesel was achieved in a semi-batch reactor under solvent-free conditions, after 9 h of reaction at 350 °C and 40 bar H_2_ pressure.

The selective hydrogenation of crotonaldehyde was studied by Kyriakou and co-workers [13] on recyclable PdCu single atom alloys supported on Al_2_O_3_ to elucidate the minimum number of Pd atoms required to facilitate the sustainable transformation of an α,β-unsaturated carbonyl molecule. By diluting the Pd content of the alloy, the reaction activity of Cu nanoparticles can be accelerated, enabling more time for the cascade conversion of butanal to butanol. In addition, a significant increase in the conversion rate was observed, compared to bulk Cu/Al_2_O_3_ and Pd/Al_2_O_3_ catalysts when normalizing for Cu and Pd content, respectively. The reaction selectivity over the single atom alloy catalysts was found to be primarily controlled by the Cu host surface, mainly leading to the formation of butanal; however, this occurred at a significantly higher rate than the monometallic Cu catalyst. The results demonstrated that fine-tuning the dilution of PdCu single atom alloy catalysts can leverage the activity and selectivity enhancement, and lead to cost-effective, sustainable, and atom-efficient alternatives to monometallic catalysts.

The simultaneous photocatalytic and adsorptive remediation efficiency of biochar–bismuth oxychloride (BiOCl) composites were studied for the removal of a benchmark azo anionic dye, methyl orange dye (MO) by Triantafyllidis and co-workers [14]. The composites consisting of bismuth oxyhalide nanoparticles, specifically BiOCl nanoplatelets, and lignin-based biochar were synthesized using a one-step hydrolysis. The influence of catalyst dosage, initial dye concentration, and pH on the photo-assisted removal were tested and optimized using the Box–Behnken design of response surface methodology (RSM). Under optimized conditions, 100% degradation of the MO after 60 min of light exposure was observed. Activated biochar had a positive impact on the photocatalytic performance of the BiOCl photocatalyst for removing the MO. This took place due to favorable changes in the surface morphology, optical absorption, and specific surface area and hence the dispersion of the photo-active nanoparticles, leading to more photocatalytic active sites.

Stamatis and co-workers [15] reported on the green production of few-layer bio-Graphene (bG) through liquid exfoliation of graphite in the presence of bovine serum albumin. The presence of 3–4-layer graphene was evidenced with microscopic characterization and spectroscopic techniques, which also confirmed the quality of the resulted bG, as well as the presence of bovine serum albumin on the graphene sheets. Then, and for the first time in the literature, bG was used as a support for the simultaneous covalent co-immobilization of three enzymes, namely *β*-glucosidase, glucose oxidase, and horseradish peroxidase. The three enzymes were efficiently co-immobilized on bG, demonstrating high immobilization yields and activity recoveries (up to 98.5 and 90%, respectively). Co-immobilization on bG led to an increase in apparent *K*_M_ values and a decrease in apparent *V*_max_ values, while the stability of the nanobiocatalysts prevailed compared to the free forms of the enzymes. Co-immobilized enzymes exhibited high reusability, preserving a significant part of their activity (up to 72%) after four successive catalytic cycles at 30 °C. Finally, the tri-enzymatic nanobiocatalytic system was applied in three-step cascade reactions, involving, as the first step, the hydrolysis of *p*-Nitrophenyl-*β*-D-Glucopyranoside and cellobiose. Therefore, these types of multi-enzymatic systems are promising to overcome economic and operating boundaries, such as lower losses due to diffusion limitations as well as the ability to recover and reuse the biocatalyst for successive reaction cycles that free enzymes are not capable of overcoming. The green synthesis of bG and the implementation of eco-friendly biocatalysts, such as enzymes, offer exciting possibilities, paving the way to the development of more sustainable catalysts for a variety of biological applications.

Li et al. [16] worked on enhancing the photogenerated charge separation of g-C_3_N_4_ by composing efficient heterojunctions with an additional organic constitution for solar–hydrogen conversion. Thus, g-C_3_N_4_ nanosheets were controllably modified with nano-sized poly(3-thiophenecarboxylic acid) (PTA) through in situ photopolymerization and then coordinated with Fe(III) via the –COOH groups of modified PTAs, forming an interface of tightly contacted nanoheterojunctions between the Fe(III)-coordinated PTA and g-C_3_N_4_. The resulting ratio-optimized nanoheterojunction displayed a ~4.6-fold enhancement of the visible light photocatalytic H_2_ evolution activity compared to bare g-C_3_N_4_. The improved photoactivity of g-C_3_N_4_ was attributed to the significantly promoted charge separation by the transfer of high-energy electrons from the lowest unoccupied molecular orbital (LUMO) of g-C_3_N_4_ to the modified PTA via the tight interface formed. This process depended on the hydrogen bond interaction between the –COOH of PTA and the –NH_2_ of g-C_3_N_4_, and the continuous favorable transfer to the coordinated Fe(III) with –OH for a connection with Pt as the cocatalyst. Therefore, a feasible strategy was demonstrated for solar light-driven energy production over the large family of g-C_3_N_4_ heterojunction photocatalysts with exceptional visible light activities.
